# Supplementary material for: Regulation of cellular and molecular markers of epithelial-mesenchymal transition by Brazilin in breast cancer cells
Source: PeerJ. 2024 May 9;12:e17360. doi: 10.7717/peerj.17360 (PMC11088821; doi:10.7717/peerj.17360)
Supplement: Supplemental Information 1 [file peerj-12-17360-s001.pdf]

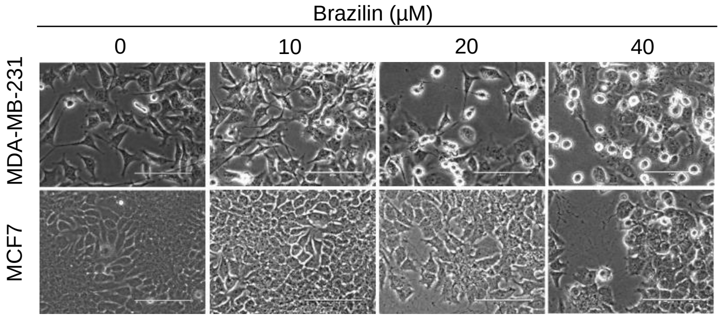

Figure 1. Brazilin induces morphological changes in MDA-MB-231, and MCF7 cells. A) Brazilin 10, 20, and 40 µM for 24 h induced morphological changes of MDA-MB-231 and MCF7 cells.

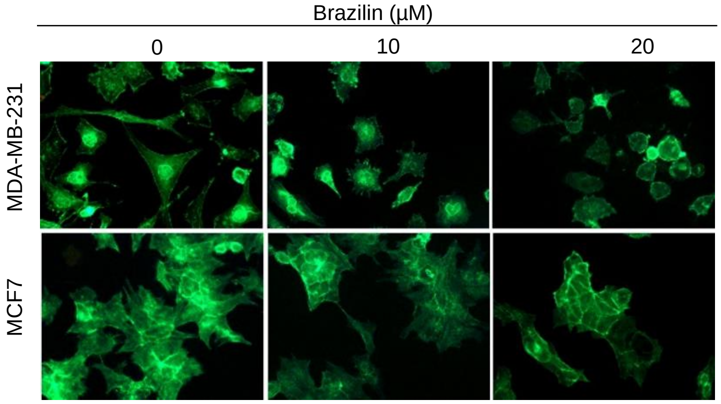

Figure 1. Brazilin induces morphological changes in MDA-MB-231, and MCF7 cells. C) Brazilin at 20 and 40 µM induces changes in actin polymerization in MDA-MB-231 and MCF7 cells.
