# Supplementary material for: Regulation of cellular and molecular markers of epithelial-mesenchymal transition by Brazilin in breast cancer cells
Source: PeerJ. 2024 May 9;12:e17360. doi: 10.7717/peerj.17360 (PMC11088821; doi:10.7717/peerj.17360)
Supplement: Supplemental Information 2 [file peerj-12-17360-s002.pdf]

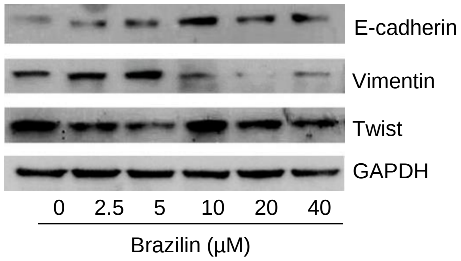

Figure 2. Brazilin regulates E-cadherin, vimentin, and Twist protein levels in MDA-MB-231 cells. Cells were treated with brazilin 0-40 µM for 24 h. A) Representative Western blot of E-cadherin (B), vimentin (C), and Twist (D) levels.

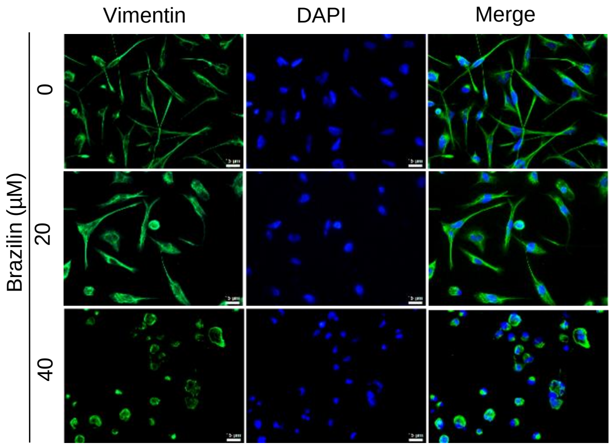

Figure 2. Brazilin regulates E-cadherin, vimentin, and Twist protein levels in MDA-MB-231 cells. Representative images of vimentin (E) by immunofluorescence assays, blue shows nuclei staining. Images were obtained at 40X objective.

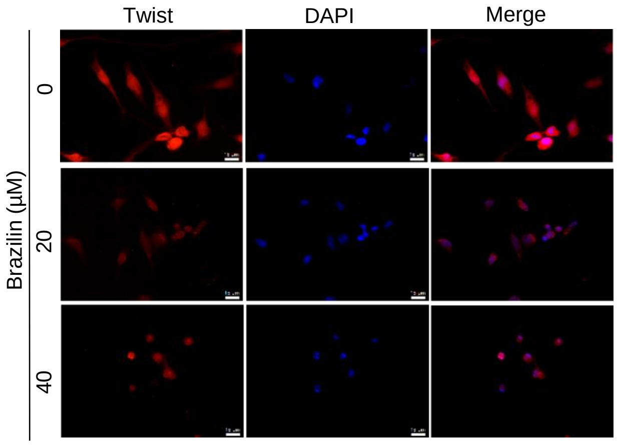

Figure 2. Brazilin regulates E-cadherin, vimentin, and Twist protein levels in MDA-MB-231 cells. Representative images of Twist (F) by immunofluorescence assays, blue shows nuclei staining. Images were obtained at 40X objective
