# Supplementary material for: Regulation of cellular and molecular markers of epithelial-mesenchymal transition by Brazilin in breast cancer cells
Source: PeerJ. 2024 May 9;12:e17360. doi: 10.7717/peerj.17360 (PMC11088821; doi:10.7717/peerj.17360)
Supplement: Supplemental Information 3 [file peerj-12-17360-s003.pdf]

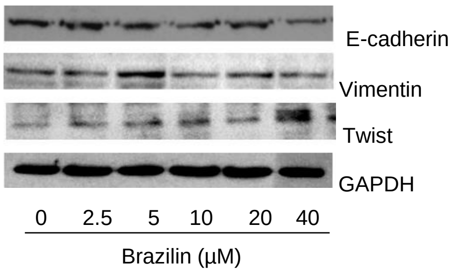

Figure 3. Brazilin regulates E-cadherin, vimentin, and Twist protein expression levels in MCF7 cells. The cell cultures were treated with brazilin 0, 2.5, 5, 10, 20, and 40 µM for 24 h. A) Representative Western blot of brazilin effect of E-cadherin (B), vimentin (C), and Twist (D) levels.

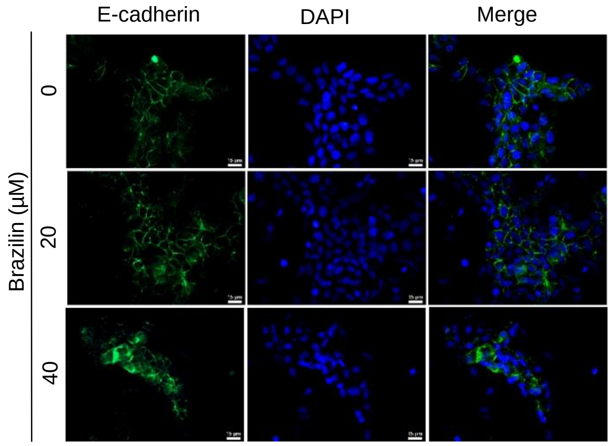

Figure 3. Brazilin regulates E-cadherin, vimentin, and Twist protein expression levels in MCF7 cells. The cell cultures were treated with brazilin 0, 2.5, 5, 10, 20, and 40 µM for 24 h. Representative images of E-cadherin (E) immunofluorescence assay, blue shows staining of nuclei. Images were obtained at 40X objective.

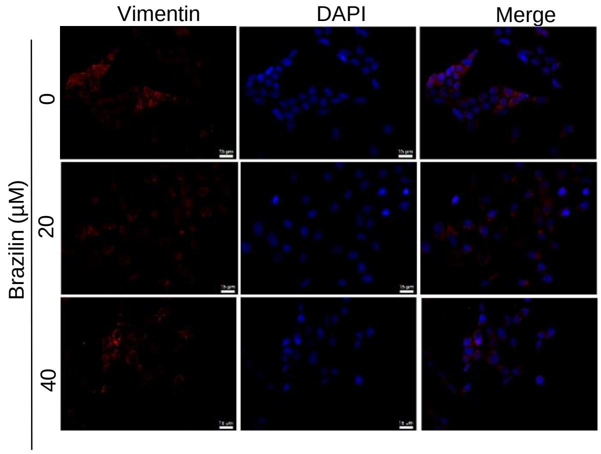

Figure 3. Brazilin regulates E-cadherin, vimentin, and Twist protein expression levels in MCF7 cells. The cell cultures were treated with brazilin 0, 2.5, 5, 10, 20, and 40 µM for 24 h. Representative images of vimentin (F) immunofluorescence assay, blue shows staining of nuclei. Images were obtained at 40X objective.
