# Supplementary material for: Regulation of cellular and molecular markers of epithelial-mesenchymal transition by Brazilin in breast cancer cells
Source: PeerJ. 2024 May 9;12:e17360. doi: 10.7717/peerj.17360 (PMC11088821; doi:10.7717/peerj.17360)
Supplement: Supplemental Information 4 [file peerj-12-17360-s004.pdf]

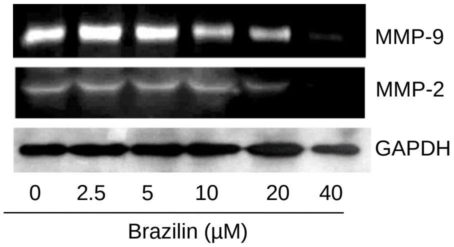

Figure 4. Brazilin decreases MMP-2 and MMP-9 secretion and invasion of MDA-MB-231 cells. A) Zymography assays of MDA-MB-231 cells treated with brazilin 0, 2.5, 5, 10, 10, 20, and 40 µM for 24 h, corresponding to MMP-9 (92 kDa) and MMP-2 (72 kDa) degradation bands.

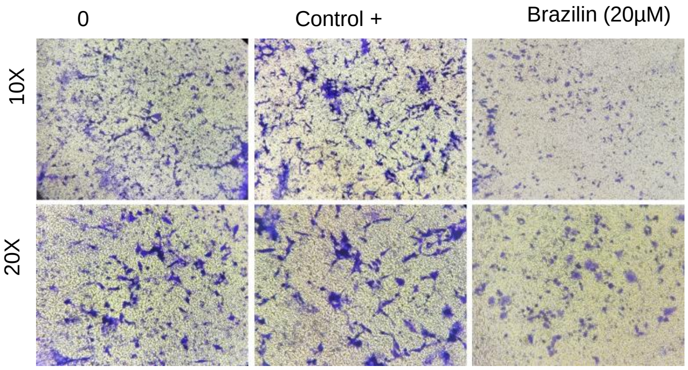

Figure 4. Brazilin decreases MMP-2 and MMP-9 secretion and invasion of MDA-MB-231 cells. D) Representative brightfield microscopy images of invasion assays of MDA-MB-231 cells treated with 0, the positive control (medium supplemented with 1% SFB, and brazilin 20 mM for 24 h.
