# Supplementary material for: Regulation of cellular and molecular markers of epithelial-mesenchymal transition by Brazilin in breast cancer cells
Source: PeerJ. 2024 May 9;12:e17360. doi: 10.7717/peerj.17360 (PMC11088821; doi:10.7717/peerj.17360)
Supplement: Supplemental Information 5 [file peerj-12-17360-s005.pdf]

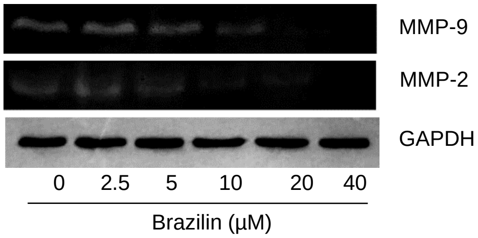

Figure 5. Brazilin decreases MMP-2 and MMP-9 secretion and invasion of MCF7 cells. A) Zymography assays of MCF7 cells treated with brazilin 0, 2.5, 5, 10, 20, and 40 µM for 24 h, corresponding to degradation bands of MMP-9 (92 kDa) and MMP-2 (72 kDa).

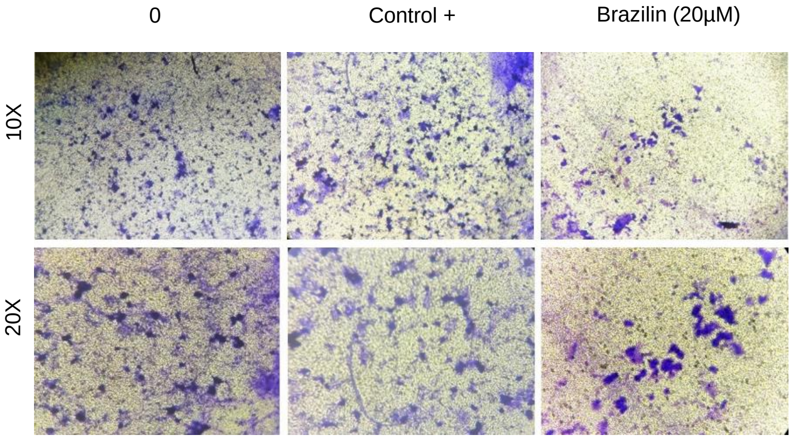

Figure 5. Brazilin decreases MMP-2 and MMP-9 secretion and invasion of MCF7 cells. D) Representative brightfield microscopy images of invasion assays of MCF7 cells, the positive control (medium supplemented with 1% SFB), and 20 µM brazilin for 24 h.
