# Supplementary figures and images for: Regulation of cellular and molecular markers of epithelial-mesenchymal transition by Brazilin in breast cancer cells
Source: PeerJ. 2024 May 9;12:e17360. doi: 10.7717/peerj.17360 (PMC11088821; doi:10.7717/peerj.17360)

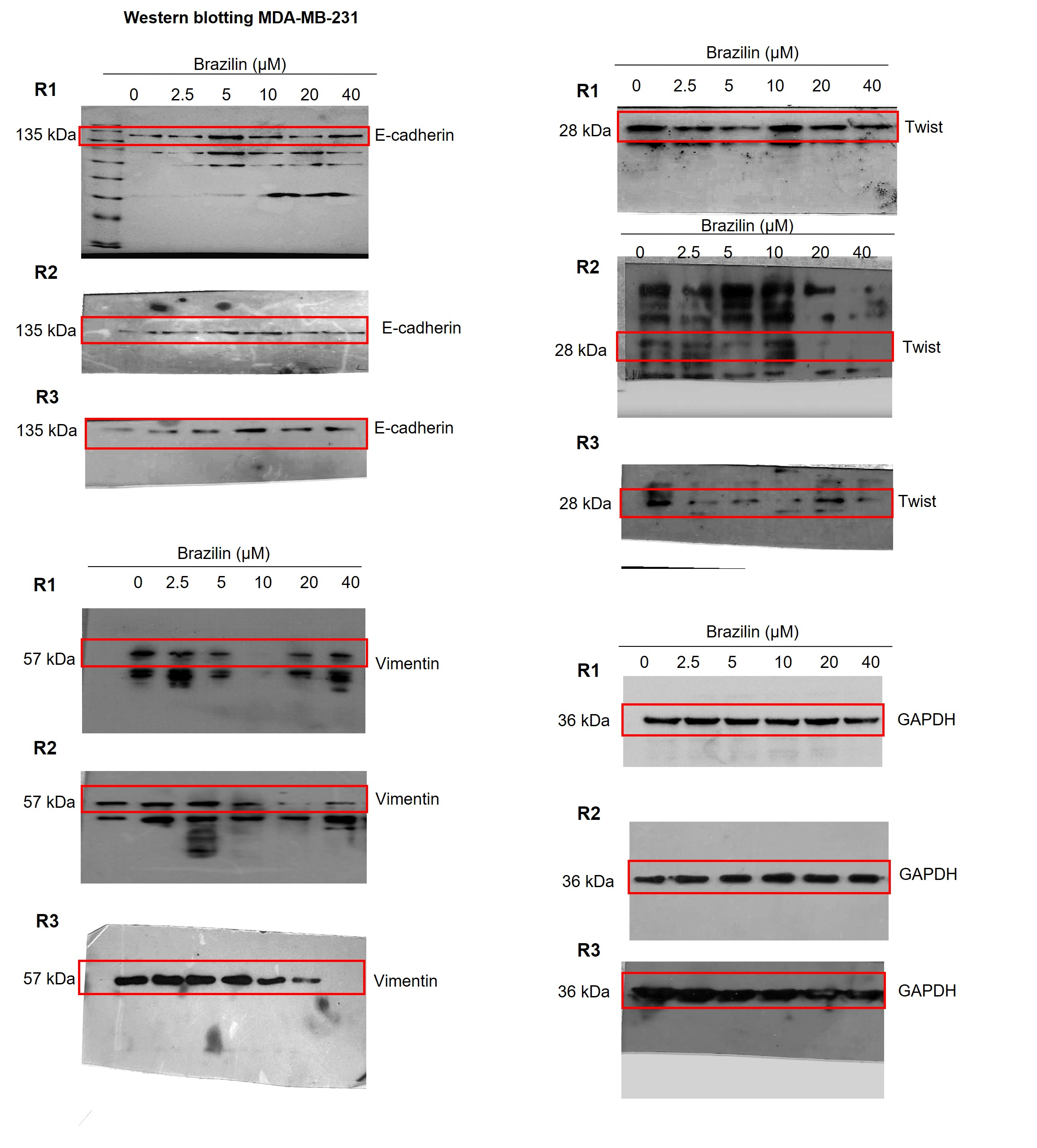

Supplement: Supplemental Information 6 — Independent replicates of the effect of Brazilin on E-cadherin, vimentin and Twist levels. [file peerj-12-17360-s006.png]

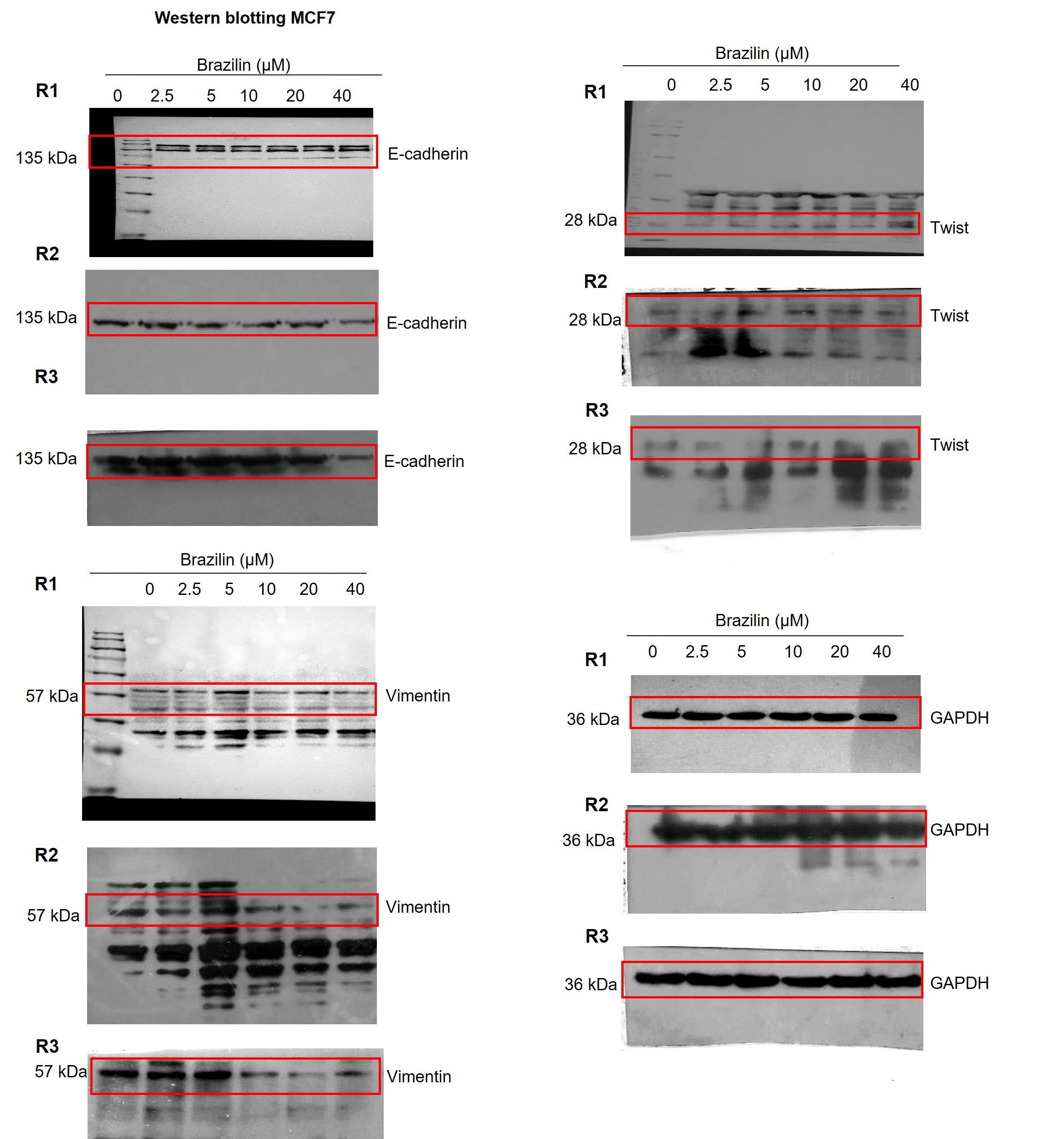

Supplement: Supplemental Information 7 — Independent replicates of the effect of Brazilin on E-cadherin, vimentin and Twist levels. [file peerj-12-17360-s007.png]

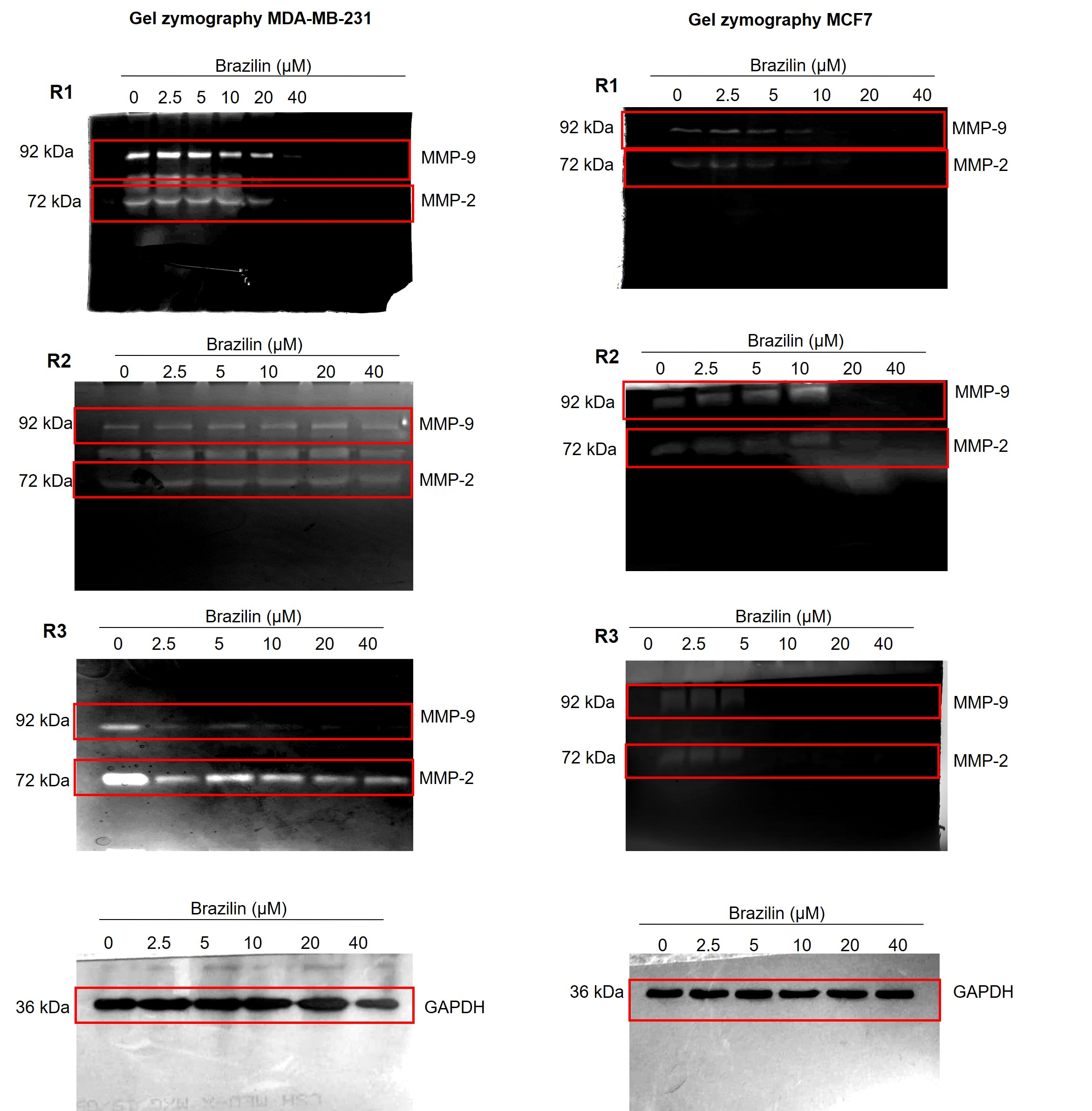

Supplement: Supplemental Information 8 — Independent replicates of the effect of Brazilin on MMP-2 and MMP-9 secretion in MDA-MB-231 and MCF7 cells. [file peerj-12-17360-s008.png]

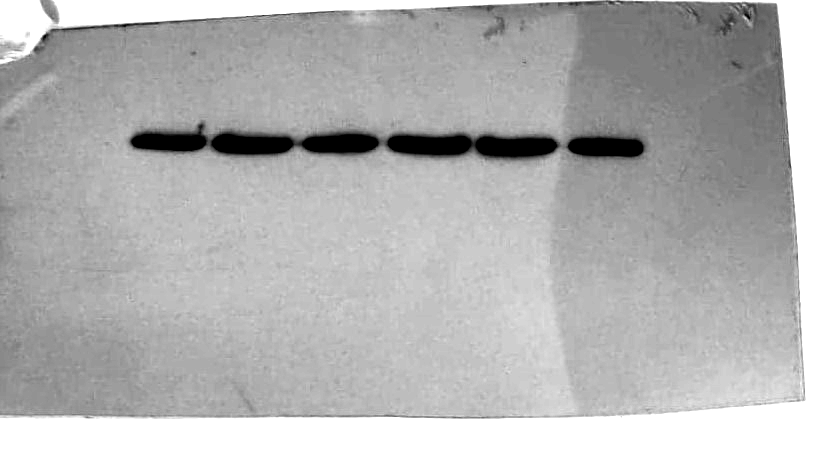

Supplement: Supplemental Information 10 — compendium of compressed images of western blots and gelatin zymography by triplicate. [file peerj-12-17360-s010.zip › Western blot MCF7/GAPDH MCF7/R1.tif]

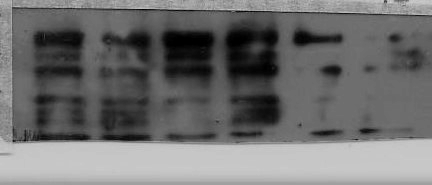

Supplement: Supplemental Information 10 — compendium of compressed images of western blots and gelatin zymography by triplicate. [file peerj-12-17360-s010.zip › Western blot MDA-MB-231/Twist MDA-MB-231/R2.tif]

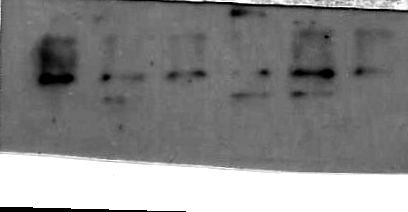

Supplement: Supplemental Information 10 — compendium of compressed images of western blots and gelatin zymography by triplicate. [file peerj-12-17360-s010.zip › Western blot MDA-MB-231/Twist MDA-MB-231/R3.tif]

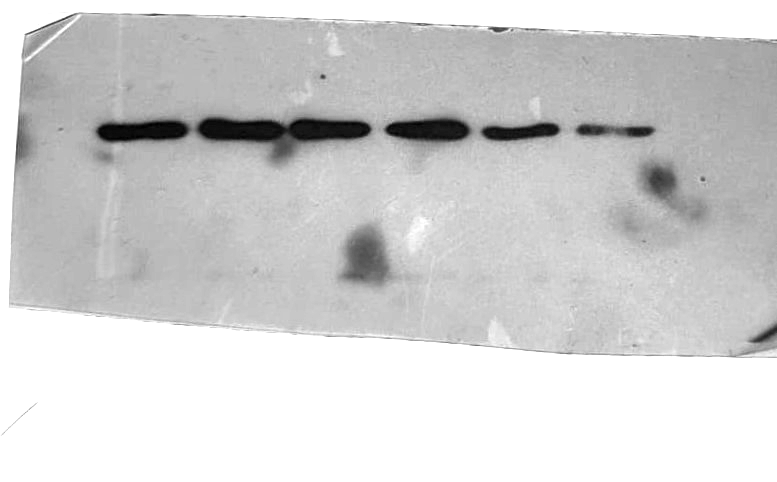

Supplement: Supplemental Information 10 — compendium of compressed images of western blots and gelatin zymography by triplicate. [file peerj-12-17360-s010.zip › Western blot MDA-MB-231/Vimentin MDA-MB-231/R3.tif]

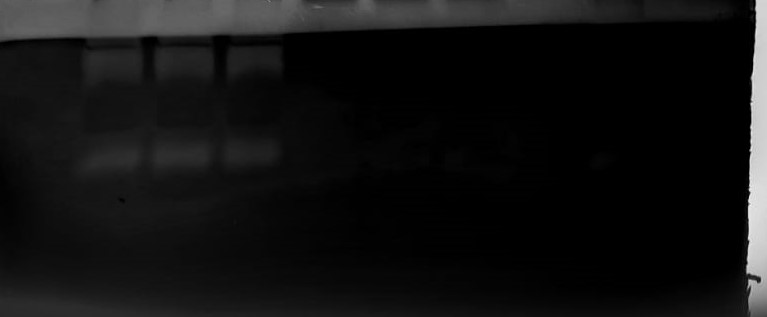

Supplement: Supplemental Information 10 — compendium of compressed images of western blots and gelatin zymography by triplicate. [file peerj-12-17360-s010.zip › Zimography MCF7/R3.tif]
